# Preparation and Characterization of New Liposomes. Bactericidal Activity of Cefepime Encapsulated into Cationic Liposomes

**DOI:** 10.3390/pharmaceutics11020069

**Published:** 2019-02-06

**Authors:** Maria Luisa Moyá, Manuel López-López, José Antonio Lebrón, Francisco José Ostos, David Pérez, Vanesa Camacho, Irene Beck, Vicente Merino-Bohórquez, Manuel Camean, Nuria Madinabeitia, Pilar López-Cornejo

**Affiliations:** 1 Department of Physical Chemistry, University of Seville, 41012 Seville, Spain; moya@us.es (M.L.M.); lebronjunior@hotmail.com (J.A.L.); fraostmar@alum.us.es (F.J.O.); david_perezalf@hotmail.com (D.P.); vane_farma@hotmail.com (V.C.); irenebeckdiaz93@gmail.com (I.B.); 2 Departament of Chemical Engineering, Physical Chemistry and Material Science. Faculty of Experimental Sciences, University of Huelva, Campus El Carmen, E-21071 Huelva, Spain; manuel.lopez@diq.uhu.es; 3 Departament of Microbiology, Faculty of Biology, University of Sevilla, 41012 Seville, Spain; vicenmb@hotmail.com (V.M.-B.); manuel.camean.sspa@juntadeandalucia.es (M.C.); 4 Hospital Pharmacy Area, University Hospital Virgen Macarena, 41009 Seville, Spain; nuria@us.es

**Keywords:** cefepime, liposome, bactericidal activity, zeta potential, encapsulation, surfactant.

## Abstract

Cefepime is an antibiotic with a broad spectrum of antimicrobial activity. However, this antibiotic has several side effects and a high degradation rate. For this reason, the preparation and characterization of new liposomes that are able to encapsulate this antibiotic seem to be an important research line in the pharmaceutical industry. Anionic and cationic liposomes were prepared and characterized. All cationic structures contained the same cationic surfactant, *N*,*N*,*N*-triethyl-*N*-(12-naphthoxydodecyl)ammonium. Results showed a better encapsulation-efficiency percentage (EE%) of cefepime in liposomes with phosphatidylcholine and cholesterol than with 1,2-dioleoyl-*sn*-glycero-3-phosphoethanolamine (DOPE). The presence of cholesterol and the quantity of egg-yolk phospholipid in the liposome increased the encapsulation percentage. The bactericidal activity against *Escherichia coli* of cefepime loaded into liposomes with phosphatidylcholine was measured. The inhibitory zone in an agar plate for free cefepime was similar to that obtained for loaded cefepime. The growth-rate constant of *E. coli* culture was also measured in working conditions. The liposome without any antibiotic exerted no influence in such a rate constant. All obtained results suggest that PC:CH:12NBr liposomes are biocompatible nanocarriers of cefepime that can be used in bacterial infections against *Escherichia coli* with high inhibitory activity.

## 1. Introduction

**Figure 1:**
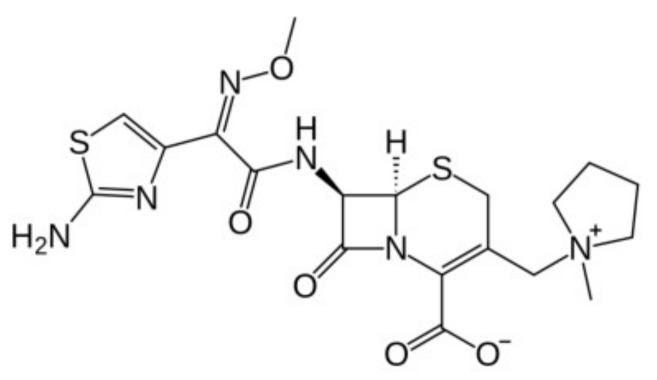
Cefepime structure.

Cefepime is a β-lactam antibiotic of the fourth generation, belonging to the cephalosporin group, which is administered by infusion or by intravenous injection. This antibiotic shows a zwitterionic structure at the determined pH values due to the presence of a charged *N*-methyl-pyrrolidine substitution at the three-cephem moiety, and a carboxyl group at the same ring (see Figure 1) [1].

The β-lactam ring of the antibiotic suffers degradation at body temperature, not being stable for 24 h in the organism [2]. This low half-life time means that continuous infusions need to be administered to the patient in order to maintain drug concentrations higher than the MIC throughout dosing intervals.

The low permeability shown by the outer membrane of Gram-negative bacteria, as well as the active efflux systems in the inner membrane generated by the pathogens to protect their intracellular functions, are known. However, the zwitterionic character of cefepime eases passage through the protein channels of the outer membrane, showing a broad spectrum against Gram-negative bacteria [3,4,5], and also good activity versus Gram-positive micro-organisms [6]. Alone, combined, or in a rotatory treatment, cefepime seems to be efficient against several bactericidal infections [7,8,9,10].

In spite of good activity versus different pathogens, the use of cefepime provokes side effects. In fact, this antibiotic shows high neurological toxicity, producing confusion, myoclonia, seizures, and nonconvulsive status epilepticus in patients with distinct pathologies (for example, brain disorders, febril neutropenia, and severe renal dysfunction) [11,12,13,14]. The use of high concentrations of this antibiotic provoked a large percentage of sepsis-related mortality [15]. The FDA indicated in 2012 that several instances of nonconvulsive status epilepticus could be associated with the use of this antibiotic when nonappropriate doses are administrated to the patient. Recently, Isitan et al. published a review about cases of cefepime-induced neurotoxicity [16].

In order to increase the short half-life time of this antibiotic and minimize its side effects, nanocarriers can be used to encapsulate it. In this sense, liposomes are artificial vesicles of different size (50–500 nm) formed by concentric layers of phospholipids around an aqueous core [17]. According to the number of formed concentric lipidic membranes, uni- or multillamellar liposomes can be prepared. The presence of hydrophilic/hydrophobic zones in the same structure permits the use of liposomes as vehicles to transport pharmaceutical agents with diverse lipophilicity [18].

Torres et al. [19] prepared liposomal formulations for cefepime and ceftazidine by using phosphatidylcholine, cholesterol, and α-tocopherol (vitamin E) with mass ratios of 4:1:0.002 and 8:1:0.005, following the lipid film hydration method. Obtained encapsulation-efficiency percentages were about 2% and 6% for cefepime and ceftazidine, respectively. Results of antibacterial activity of both liposomal cefepime and ceftazidime against *P. aeruginosa* indicated that liposomal formulations can be an effective alternative in the treatment of infections caused by these micro-organisms, and a valid approach against the development of bacterial resistance (an added problem to the use of high doses of antibiotics in patients).

**Figure 2:**
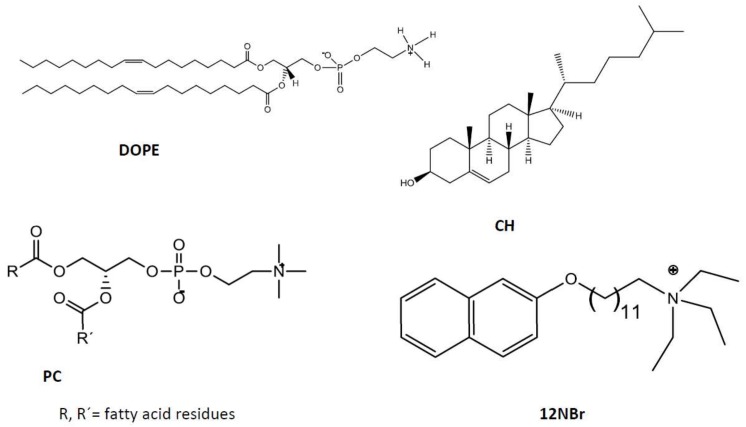
Structure of lipids and cholesterol used in this work.

A multidisciplinary study was done to prepare and characterize liposomes by using mixtures of diverse biocompatible and nontoxic lipids with the goal of encapsulating cefepime. Results showed higher encapsulation efficiency (EE%) with cationic liposomes than with the anionic ones. The addition of cationic surfactant *N*,*N*,*N*-triethyl-*N*-(12-naphthoxy dodecyl)ammonium bromide (12NBr) increased EE%. On the other hand, cefepime loaded into cationic liposomes showed high bacterial-inhibition activity against *E. coli*. The release of the antibiotic from the liposomes was also studied. The structures of the used lipids (DOPE, PC, CH, and 12NBr) are shown in Figure 2.

## 2. Materials and Methods

### 2.1. Chemicals

l-α-phosphatidylcholine (PC) from egg yolk, 1,2-dioleoyl-*sn*-glycero-3-phosphoethanolamine (DOPE), and cholesterol (CH) were purchased from Sigma-Aldrich and used as supplied. The *N*,*N*,*N*-triethyl-*N*-(12-naphthoxydodecyl)ammonium surfactant (12NBr) was synthesized and characterized as reported in Reference [20]. Cefepime (see Figure 1) and chloroform (analytical grade) were from Normon (Madrid, Spain) and Merck (Mollet del Vallès, Barcelona, Spain), respectively, and used as received.

All solutions were prepared with distillated and deionized water obtained from a Millipore Milli-Q system (Fisher Scientific Company, Ottawa, ON, Canada), with a conductivity value lower than 10^−6^ Sm^−1^. The pH of the solutions was maintained constant at a value of 7.4 by using a HEPES (4-(2-hydroxyethyl)piperazine-1-ethanesulfonic acid, from Sigma-Aldrich) buffer (I = 0.01 mol dm^−3^). Measurements were done at 303.0 ± 0.1K.

### 2.2. Liposome Preparation

Cationic and anionic liposomes were prepared by using the lipid film hydration method [20,21] with diverse modifications depending on the used lipids. In order to prepare lipid bilayers, lipids were first dissolved in chloroform. The lipid solution was maintained for 2 min in an ultrasound machine (J P Selecta Ultrasons system, 200 W and 50 kHz) at room temperature (for DOPE liposomes) or 38 °C (for PC liposomes). Fluidity of the lipid bilayer depends on the transition temperature [22,23] of the lipid, that is, the temperature required to induce a change in the lipid physical state from a gel phase (ordered state with fully extended hydrocarbon chains) to a liquid crystalline phase (a more disordered and fluid state, in which hydrocarbon tails are randomly oriented) [24].

The organic solvent of the obtained clear lipid solution was evaporated by using a rotary evaporator for 45 min at 30 °C, and obtaining the lipid bilayer, which was stored at −81 °C for at least 24 hours in order to avoid degradation of the phospholipids.

After 24 h, the lipid bilayer was hydrated with 2 ml of an aqueous solution of HEPES 10 mM (pH = 7.4) containing the desired quantity of cefepime. The hydration of the lipid bilayer was done in two steps. First, a hydration process of 50 min in 10 cycles for DOPE (25 min in 5 cycles for PC) of vigorous shaking, a vortex process (3 min) and an ultrasound process (2 min) were alternately carried out. The second hydration step consisted of a vigorous vortexed process of 2 h for DOPE (1 h for PC). The formed liposomes were multillamellar and showed high polydispersity. In order to generate uniform populations of unilamellar liposomes with sizes lower than 200 nm, the extrusion of the liposome solutions was done by using a miniextruder from Avanti Polar Lipids and polycarbonate membranes of diameters of 100 and 250 nm from Whatman. All liposome solutions were extruded 10 times.

The composition of the cationic liposomes is expressed in molar fraction, *α*, defined as the molar ratio between the cationic lipid and the total lipid (see Equation (1)).

(1) $$\alpha =\;\frac{n_{+}}{n_{o}+\;n_{+}},$$

*n*_+_ and *n*_o_ being the molar concentration of the cationic and neutral lipid, respectively. All concentrations refer to the total volume of solution.

The composition (mass ratio) of the different prepared liposomes is shown in Table 1.

### 2.3. Absorbance Measurements

Absorbance spectra were performed to check stability, and characterize and quantify the concentration of the different cefepime solutions. Absorption spectra were run in a Cary 500 SCAN UV-vis-NIR (Varian). Quartz cells of 10 mm path length were used. Data were collected every 1 nm, and spectra were recorded in a wavelength range from 200 to 400 nm. Standard quartz cuvettes of 10 mm were used. Blank was performed with a HEPES 10 mM solution. Temperature was maintained constant at 303.1 ± 0.1 K.

### 2.4. Zeta-Potential Measurements

Zeta-potential (ζ) experiments were carried out with a Zetasizer Nano ZS Malvern Instrument Ltd (Malver, Worcestershire, UK). This parameter measures the electrophoretic mobility of the sample from the velocity of the particles using a Laser Doppler velocimeter (LDV, (Malver, Worcestershire, UK). A DTS1060 polycarbonate capillary cell was used at 303.1 ± 0.1 K.

### 2.5. Dynamic Light-Scattering Measurements (DLS)

The size and the polydispersity index of the different liposomes were obtained from DLS measurements by using a Zetasizer Nano ZS Malvern Instrument Ltd (UK). Samples were illuminated with a laser at a fixed detection arrangement of 90° to the center of the cell area, and fluctuations in the intensities of the scattered light were analyzed. The obtained results were the average of 10 measurements.

### 2.6. Encapsulation-Efficiency Measurements

Drug encapsulation efficiency was measured by using a dialysis method. First, 500 μl of drug-loaded liposome was added to a Pur-A-Lyzer Midi 1000 Dialysis Kit (MWCO 1 kDa) from Sigma-Aldrich (Rancho Dominguez, CA, USA), a dialysis tubing of a molecular weight cut-off = 12 kDa from Sigma-Aldrich was also used and results were not different). The dialyzer was sunk into a beaker containing 80 mL of HEPES 10 mM. An aliquot of 2 mL from the buffer deposited on the beaker was taken each 15 min. The quantification of the loaded antibiotic was carried out by UV-vis spectroscopy at 258 nm. The taken aliquots were replaced each time by an equal volume of buffer in order to keep the buffer volume in the beaker constant. Dialysis was followed for a period time of 24 h at least. The EE% was obtained by using Equations (2) and (3). Each measurement was carried out in triplicate.

(2) $$\mathrm{EE}\;\%=\frac{{\left[\mathrm{antibiotic}\right]}_{m}}{{\left[\mathrm{antibiotic}\right]}_{\mathrm{total}}}\;\times \;100,$$

(3) $${\left[antibiotic\right]}_{m}=\;{\left[antibiotic\right]}_{total}-\;{\left[antibiotic\right]}_{\mathrm{buffer}},$$

[antibiotic]_m_, [antibiotic]_buffer_, and [antiobiotic]_total_ are the drug concentration encapsulated in the liposomes, the concentration in the buffer solution, and the total concentration added to the liposomal solution, respectively. All concentrations refer to the total solution volume.

The used cefepime concentration in the measurements was in the range of 20–120 µg/mL. The quantification of the loaded antibiotic was carried out by UV-vis spectroscopy at 258 nm.

In order to avoid the degradation of cefepime in our measurements, these dialysis studies were done in darkness and at 4 °C.

### 2.7. Cefepime Release

The release of the antibiotic from the PC:CH:12NBr liposome was obtained by using the same dialysis method utilized in the encapsulation-efficiency measurements. In this case, dialysis was carried out at 37 °C. Aliquots of 2 mL were taken at a certain time interval in order to know the concentration of cefepime releasing from the liposome. The quantification of the loaded antibiotic was carried out by UV-vis spectroscopy at 258 nm.

### 2.8. In Vitro Antibacterial Activity

Antibacterial activity of cefepime-loaded liposomes was tested against Gram-negative bacteria *Escherichia coli* (*E. coli*) using the well-diffusion method on agar and a spectroscopic method in solution.

*Escherichia coli* strain CECT 515 [25] was incubated in a Luria-Bertani medium [26] overnight at 37 °C with continuous shaking at 180 rpm. This culture reached a cell concentration of approximately 10^9^ cells/mL, and the *E. coli* culture was diluted 10 times (~10^8^ cells/mL). Then, 200 μL was spread from this diluted culture on LB plates containing 2% agar. Each LB plate contained about 2 × 10^7^ *E. coli* cells.

In a first qualitative study, antimicrobial activity was evaluated as follows: 100 μL of cefepime-free, PC liposomes, and cefepime-loaded liposomes was uniformly added to disks of an extra-thick blot filter paper from Bio-Rad, sufficiently separated, and put on the agar plates. The plates were incubated at 37 °C for 24 h. After the incubation period, the diameters of the growth-inhibition zones were measured. Used cefepime concentrations were 120, 70, 50, and 20 μg/mL.

Second, a quantitative study was followed to calculate the inhibition that the cefepime loaded into the liposomes exerted on the growth of *E. coli* bacterial suspensions in liquid cultures. Both cell and cefepime concentrations used in this quantitative method were the same as those used in the agar plates (about 2 × 10^7^ of *E. coli* cells and 20 μg/mL of the antibiotic). Microbial growth was evaluated by measuring the absorbance of the culture at 600 nm in a Synergy HTX Multi-Mode Microplate Reader from Biotek with the use of 96-well plates. Absorbance measurements were done at 37 °C.

All measurements were repeated 3 times.

## 3. Results and Discussion

Anionic and cationic liposomes were prepared and characterized. *ζ* is the potential in the sliding plane of colloidal particles that is closely related to the characteristics of electric double layers in these nanostructures. Table 1 collects zeta-potential values obtained for the prepared diverse liposomes. As can be seen, *ζ* value slightly depends on the quantity of added lipid. In the case of the anionic liposomes (PC:CH), an increase in lipid concentration does not exert any influence on the zeta-potential value of such structures. In the case of cationic liposomes (PC:CH:12NBr), *ζ* value decreases with lipid mass at constant masses of both cholesterol and cationic surfactant (samples C and E in Table 1). The same variation was observed comparing samples D and F in Table 1. This behavior takes place due to the slight negative charge of the used phosphatidylcholine in this work. However, no dependence of zeta potential was observed on the α parameter (see Equation (1)).

With respect to liposomes formulated with DOPE and cationic surfactant 12NBr, no dependence was observed with molar fraction α, either. If different cationic formulations prepared with DOPE or PC are compared, results show higher zeta-potential values for liposomes with PC than those with DOPE for the same α molar fractions. All these trends are difficult to explain. The different lipid structures or packing of each lipid bilayer influences zeta-potential values.

**Figure 3:**
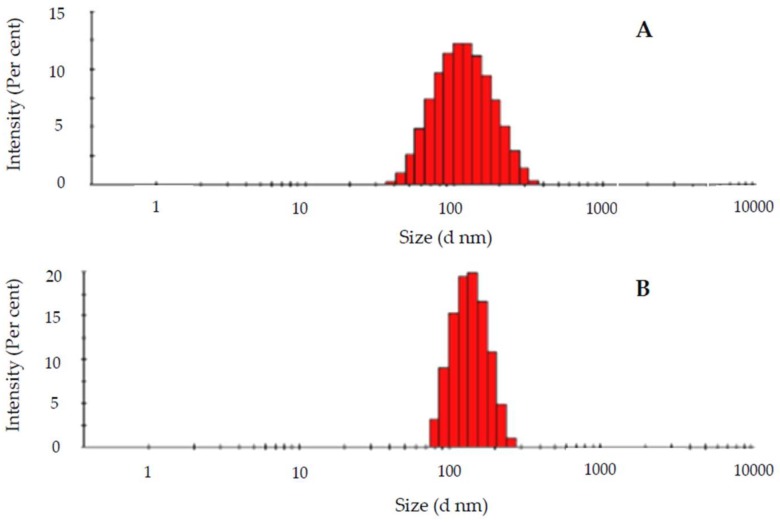
Intensity/size distribution of a liposome containing l-α-phosphatidylcholine (PC), cholesterol (CH), and *N*,*N*,*N*-triethyl-*N*-(12-naphthoxy dodecyl)ammonium bromide (12NBr) (Sample C in Table 1) in the (**A**) absence and (**B**) presence of cefepime.

**Figure 4:**
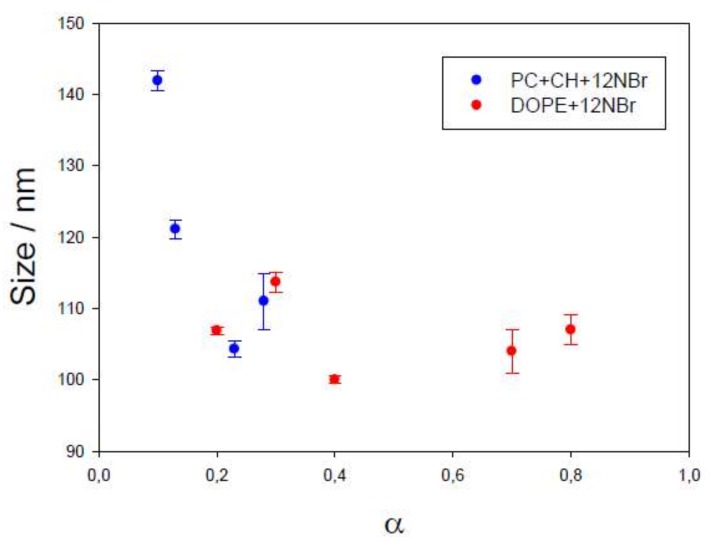
Dependence of liposome size on the α parameter for different liposomes. Error bars represent standard deviation in each α value (*n* = 5).

Dynamic light scattering (DLS) evaluates the hydrodynamic radius of particles in a solution. Size data obtained for the liposomes prepared in this work, as well as the polydispersity obtained, are collected in Table 1. According to the results, the different liposomes had similar sizes, about 100 nm. This is an excellent size value for a drug nanocarrier to be intravenously administrated [27]. Sizes do not practically depend on the character of the lipid (PC or DOPE, see Figure 1), or on the presence or absence of the antibiotic, as observed in Figure 3. However, size depends on the α parameter, that is, on the fraction of cationic surfactant, increasing for lower α values (see Figure 4).

The polydispersity index (Pdi) determines the homogeneity grade in the size of a particle solution. A sample is considered homogeneous when Pdi is lower than 0.3 [28]. As is shown in Table 1, the obtained Pdi values are ≤0.3, demonstrating the homogeneity of the prepared liposomes with respect to their sizes. 

**Figure 5:**
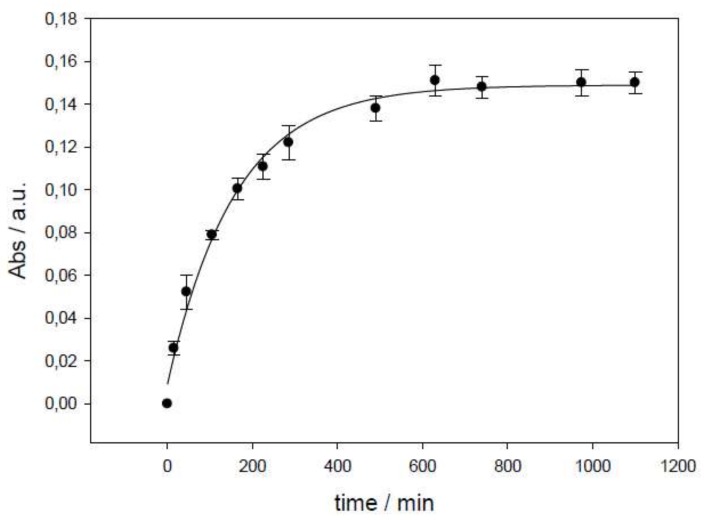
Absorbance dependence on time in a dialysis process (liposome PC + CH, Sample A). Error bars represent standard deviation in each time value (*n* = 5).

The prepared liposomes were used as cefepime nanocarriers. Obtained encapsulation-efficiency values from dialysis are shown in Table 1. Figure 5 shows an example of the absorbance variation observed in the dialysis process.

Higher encapsulation efficiency takes place in liposomes with PC and CH in comparison with those containing DOPE as a lipid. It is known that cholesterol affects the mechanical properties of lipid membranes. It increases their mechanical strength, influences membrane elasticity, and increases packing density through the ordering and condensing effects [29,30,31]. In fact, the presence of this sterol generates a less-permeable membrane. This explains why encapsulation efficiency would be higher with liposomes in the presence of cholesterol. On the other hand, the EE% is also augmented by increasing total lipid concentration (PC and 12NBr) with respect to CH quantity in these cationic liposomes (see Table 1, Samples C–F).

The EE% value of cefepime does not show any relation to the liposome size (see Table 1). However, there is an augmentation of encapsulation efficiency with zeta potential (a more positive superficial charge seems to favor the encapsulation process), although the percentage of cholesterol could also exert some influence. 

Our results are in accordance with those obtained by other authors [19] with similar liposomes (PC + CH + α-tocopherol, with mass ratios 8:1:0.005 and 4:1:0.0022). Torres et al. obtained an EE% value of 2%, while a value of about 3% was obtained in this work with the same mass ratio of PC and CH. At least initially, the presence of 12NBr (or α-tocopherol) seems to not exert any influence. However, an increase in liposome charge due to the presence of a cationic surfactant demonstrated an increase in EE% value. It is worth noting that an increase of both the PC and CH percentage, and the liposome charge, augmented cefepime’s EE%. Therefore, liposome composition was optimized in this work.

**Figure 6:**
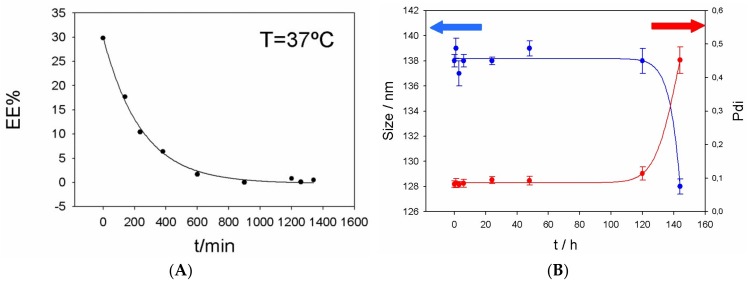
(**A**) Release of cefepime from liposome PC:CH:12NBr (Sample C) at 37 °C. (**B**) Size and Pdi of liposome PC:CH:12NBr (Sample C) at different times.

The release of cefepime from PC:CH:12NBr liposomes was also measured (see Figure 6). The variation of EE% with time was observed and it is shown in Figure 6A. Cefepime takes about 10 hours to leave the liposome at 37 °C, while the liposome takes about five days to change its size and Pdi at the same temperature (see Figure 6B).

**Figure 7:**
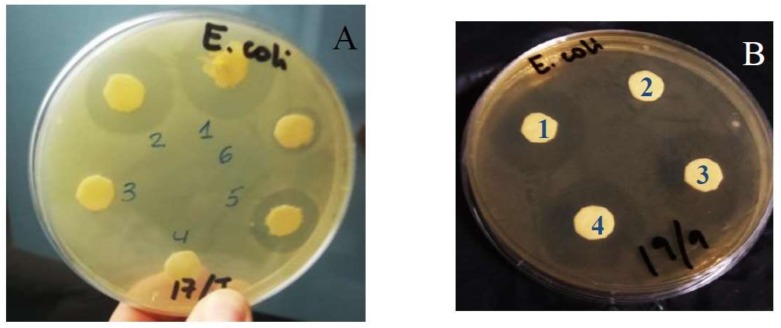
Zones of inhibition produced by cefepime after 24 h of incubation at different antibiotic concentrations and 37 °C: (**A**) [Cefepime]_free_ = 120 µg/mL (1); [Cefepime]_encapsulated_ = 120 µg/mL (2), 50 µg/mL (5), 20 µg/mL (6). Disks 3 and 4 only contain liposomes. Sample D in Table 1 was used in this assay. (**B**) [Cefepime]_free_ = 70 µg/mL (1); [Cefepime]_encapsulated_ = 120 µg/mL (3), 70 µg/mL (4). Disk 2 contains liposome without cefepime. Sample F in Table 1 was used in this assay.

It is known that cefepime shows high activity against many Gram-negative micro-organisms [32,33]. The bacterial activity of cefepime loaded into liposomes was tested against the *E. coli* bacterium through in vitro measurements in PC:CH:12NBr liposomes. Figure 7 shows the qualitative results obtained from agar well-diffusion assays. Significant inhibitory activity against *E. coli* was observed. Data for free cefepime are similar to those obtained with liposomes loaded with cefepime, always for the same concentration of antibiotics (see Disks 1 and 4 in Figure 7B). The liposome without antibiotic did not show any inhibitory activity against such bacteria (see Disks 3 and 4 in Figure 7A, and Disk 2 in Figure 7B).

**Figure 8:**
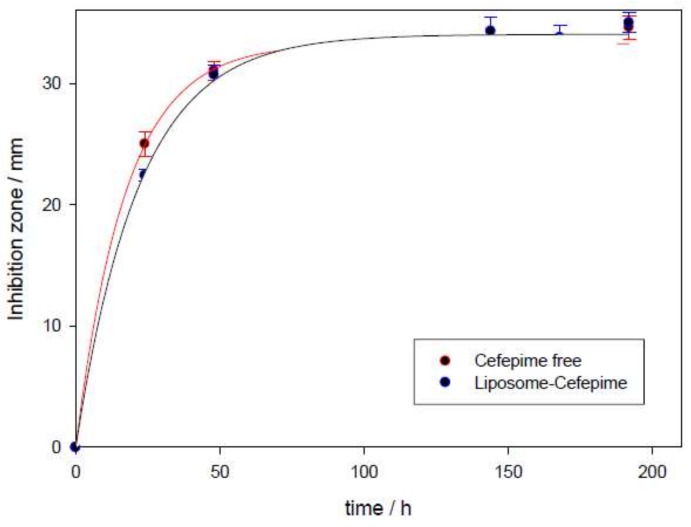
Dependence of inhibition zone with time in the presence and absence of PC:CH:12NBr liposomes at 37 °C. Error bars represent standard deviation in each time value (*n* = 3).

The zone of inhibition increased with respect to cefepime concentration (see Disks 1, 2, 5, and 6 in Figure 7A). This zone also shows dependence on incubation time for these drug carriers (see Figure 7 and Figure 8). A plateau was observed in the plot of the inhibitory zone versus time. Although inhibition zones provoked by cefepime are similar in the presence and absence of liposome, the obtained plateau appeared a little later for the antibiotic encapsulated into liposomes than for the free antibiotic. This is related to the release of the antibiotic from the nanocarrier. This result suggests that PC:CH:12NBr liposomes are good drug carriers, and show good effectiveness in killing *E. coli* bacteria.

**Figure 9:**
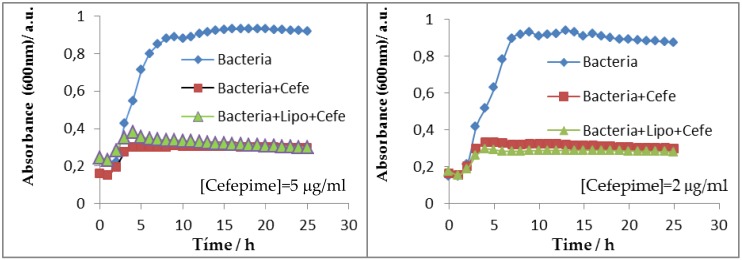
Growth inhibition of *E. coli* in the absence and presence of cefepime (free and encapsulated in PC:CH:12NBr liposomes) at different antibiotic concentrations.

**Figure 10:**
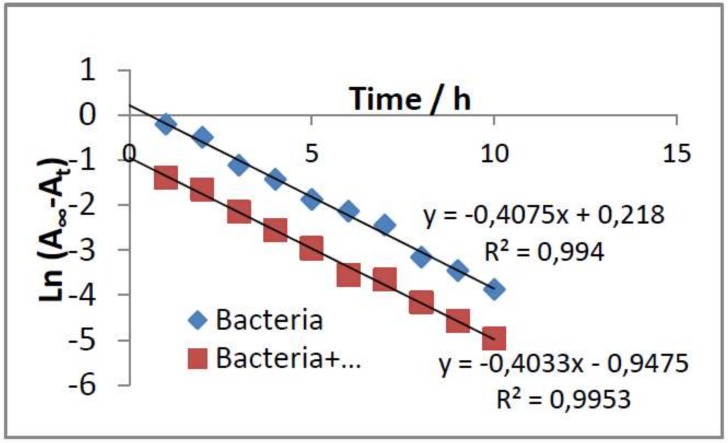
Determination of growth-rate constant for *E. coli* by using Equation (4).

Inhibitory activity against *E. coli* was also studied in solution. Absorbance corresponding to bacterial population was measured in the presence and absence of the cefepime antibiotic and/or the PC:CH:12NBr liposome. Figure 9 shows the absorbance results measured at 600 nm at different times. As can be seen, cefepime inhibited the growth of *E. coli*. The inhibition was the same for both free and encapsulated antibiotic for cefepime concentrations equal to 2 and 5 µg/mL. The liposome exerted no dependence on bacterial growth. The growth-rate constant of the bacteria in the absence and presence of liposome was calculated (see Figure 10). Assuming that cellular growth follows a first-order kinetic, the rate constant was calculated by using Equation (4):

(4) $$Ln\left(A_{\infty }-A_{t}\right)=Ln\left(A_{\infty }-A_{o}\right)-kt,$$

where *A*_o_, *A*_∞_, and *A*_t_ represent the absorbance (optical density) of the bacterial culture at a wavelength of 600 nm at different times after incubation with the antibiotic: initial time, infinite time, and at a determined *t* time, respectively. k represents the growth-rate constant of the bacteria.

The growth-rate constants of *E. coli* in the absence and presence of the PC:CH:12NBr liposome, obtained by using Equation (4), were 0.4075 and 0.4033 h^−1^, respectively. This shows that the liposome did not inhibit the growth of *E. coli*, and confirms previous results obtained from agar well-diffusion assays.

## 4. Conclusions

The encapsulation of the β-lactam antibiotic cefepime was investigated into diverse cationic and anionic liposomes with phosphatidylcholine and DOPE as lipids. A new surfactant was added to the solution to prepare cationic liposomes. Higher encapsulation efficiency was observed for the cationic liposomes. This is an interesting result because, in general, cationic nanostructures also show good internalization into cells. However, encapsulation was also more favorable for liposomes formed by phosphatidylcholine and cholesterol.

In vitro bacterial-activity studies were carried out. Results showed an inhibitory activity of cefepime-loaded liposomes against *E. coli,* similar to that observed for free cefepime. Liposomes free of antibiotic did not show any inhibitory activity at the used concentration. In fact, the growth-rate constant of *E. coli* obtained in the presence and absence of the liposome without antibiotic was 0.4075 and 0.4033 h^−1^, respectively.

The inhibition zone was measured at different times in the presence and absence of PC:CH:12NBr liposomes. Results showed the influence of cefepime release in inhibitory activity.

The obtained results invite us to use these liposomes, and others built with different cationic biosurfactants, in further investigations with the β-lactam antibiotic meropenem to be used for parenteral administration. The low solubility of this drug and its high degradation highlight the importance of this future research.

## Figures and Tables

**Table 1:** Liposome composition (mass ratio) and characterization parameters. Encapsulation efficiency percentage (EE%) of cefepime into these liposomes. α represents molar ratio between the cationic lipid and the total lipid (Errors in EE% were always lower than 10%). Pdi: polydispersity index.

Liposomes	SAMPLE	Mass Ratio	Mol Ratio	α	ζ/mV	Size/nm	Pdi	EE%
PC:CH	A	5.3:1	2.5:1	-	−24 ± 1	117 ± 9	0.1	17
	B	1.2:1	0.6:1	-	−29 ± 1	121 ± 8	0.2	19
PC:CH: 12NBr	C	21:1:1.5	10:1:1.2	0.14	+45 ± 1	138 ± 24	0.1	27
	D	32:1:3.5	15:1:2.5	0.19	+42 ± 2	121 ± 13	0.2	43
	E	7.6:1:1.5	3.6:1:1	0.25	+54 ± 4	104 ± 12	0.1	3
	F	16:1:3.5	7.5:1:3	0.35	+48 ± 2	111 ± 14	0.2	13
DOPE:12NBr	G	1:1.6	1:2.5	0.20	+30 ± 1	107 ± 15	0.2	6
	H	1:2.8	1:4.2	0.30	+28 ± 1	114 ± 14	0.3	6
	I	1:4.4	1:6.7	0.40	+31 ± 1	100 ± 15	0.2	2
	J	1:15	1:23	0.70	+32 ± 4	140 ± 28	0.3	8
	K	1:25	1:38	0.80	+37 ± 2	107 ± 12	0.2	7
